# Phenotypic Spectrum in Three Romanian Patients with 8q23–q24 Deletions

**DOI:** 10.3390/ijms27031249

**Published:** 2026-01-27

**Authors:** Alexandru Caramizaru, Ioana Streata, Andrei Pirvu, Simona Sosoi, Andreea Dumitrescu, Mihai Cucu, Georgiana-Cristiana Camen, Daniela Vasile, Elena Braha, Anca-Lelia Riza, Amelia Dobrescu, Florin Burada

**Affiliations:** 1 Regional Center for Medical Genetics Dolj, 200642 Craiova, Romania; alexandru.crgm@gmail.com (A.C.); ioana.streata@umfcv.ro (I.S.); andrei.crgm@gmail.com (A.P.); simona.sosoi@umfcv.ro (S.S.); andreearoxanadumitrescu@gmail.com (A.D.); mihai.cucu@umfcv.ro (M.C.); buradaflorin@gmail.com (F.B.); 2 Medical Genetics Department, University of Medicine and Pharmacy of Craiova, 200349 Craiova, Romania; 3 Laboratory of Human Genetics, University of Medicine and Pharmacy of Craiova, 200349 Craiova, Romania; 4 Radiology and Medical Imaging Department, University of Medicine and Pharmacy of Craiova, 200349 Craiova, Romania; 5 Radiology and Medical Imaging Department, Emergency Clinical County Hospital of Craiova, 200642 Craiova, Romania; 6 National Neuropsychomotor Rehabilitation Center “Dr. Nicolae Robănescu”, 041408 Bucharest, Romania; danavd@gmail.com; 7 National Institute of Endocrinology “C.I. Parhon”, 011863 Bucharest, Romania; elena.braha@parhon.ro

**Keywords:** trichorhinophalangeal syndrome II, TRPS II, Langer-Giedion, 8q23–q24 deletions, *TRPS1*, microarray, MLPA

## Abstract

Trichorhinophalangeal syndrome type II (TRPS II) is a rare disease caused by a contiguous gene deletion in the 8q23.3–q24.11 region. Three genes (*TRPS1*, *RAD21*, and *EXT1*) are considered responsible for the most common clinical features, which include facial dysmorphism, ectodermal and skeletal anomalies, osteochondromas, and cognitive impairment. To date, seven patients with 8q23–q24 deletions not involving *TRPS1* have been reported, with phenotypes overlapping TRPS II. In this paper, we present clinical and genetic aspects from three non-related patients with 8q23–q24 deletions, and we review the available testing strategies for such patients and their families. The deletions harbored by these patients have been identified through microarray, with two of them also undergoing initial MLPA evaluation. The observed clinical and genetic features are heterogeneous, and generally in keeping with known associations between the three main genes from the deleted region and the clinical manifestations of TRPS II. Particularly, the deleted regions vary substantially in size, genomic coordinates, and gene content, with one not including *TRPS1*, and another, with a more distal loss, not including either *TRPS1* nor *RAD21*. By describing three new patients, we hope to enlarge the genetic and clinical landscape of TRPS II and 8q23–q24 deletions, and help identify further genotype–phenotype correlations.

## 1. Introduction

Trichorhinophalangeal syndrome type II (TRPS II, OMIM 150230), also known as Langer–Giedion syndrome (LGS), is a dysmorphic skeletal neurodevelopmental disorder resulting from a microdeletion in the 8q23.3–q24.11 region. Its clinical manifestations, encompassing distinctive facial features, ectodermal abnormalities, skeletal anomalies, osteochondromas, and an elevated risk of intellectual disability, are attributed to this contiguous gene deletion on chromosome 8, and mainly associated with three genes: *TRPS1*, *RAD21*, and *EXT1*. A related condition, trichorhinophalangeal syndrome type I (TRPS I), is caused by heterozygous pathogenic variants in *TRPS1* (OMIM 190350) [1]. Although TRPS type III (OMIM 190351) has been described as a severe form characterized by significant brachydactyly and short stature [2], it is now considered to be within the TRPS I spectrum due to its etiology involving *TRPS1* variants [3].

The prevalence of TRPS type II is unknown, with approximately 100 cases reported in the literature to date, according to Orphanet. The largest reported cohort of TRPS consisted of 103 patients, of which only 14 had TRPS II [3].

*TRPS1* encodes the zinc finger transcription factor Trps1, a protein that functions as a repressor of GATA–regulated genes while also possessing the capacity to activate transcription of specific target genes [4]. Its cellular activity is related to bone mineralization, chondrocyte differentiation, and hair follicle development, in keeping with the association with TRPS [1,4]. The *RAD21* gene product is a component of the cohesin complex with an important role in chromatid segregation [5,6], having also been linked with Cornelia de Lange syndrome 4 (OMIM 614701) [7,8], and chronic intestinal pseudo-obstruction (CIPO) [9,10]. As for *EXT1*, this gene contributes to the formation of a protein complex controlling the differentiation, ossification, and apoptosis of chondrocytes. Pathogenic variants in *EXT1* cause autosomal dominant multiple exostoses type 1 (OMIM 133700) [11].

A significant clinical overlap exists between trichorhinophalangeal syndrome type I and type II. Given that TRPS II is caused by a microdeletion, it typically presents with a broader spectrum of clinical features. Both syndromes exhibit substantial clinical heterogeneity. Furthermore, as these two TRPS variants arise from distinct types of genetic modifications, different diagnostic approaches may be considered [1].

Furthermore, 8q23–q24 deletions sparing the *TRPS1* gene also determine phenotypes overlapping TRPS II, with only seven patients described in the literature to date [5,12,13,14,15,16].

This paper aims to characterize three Romanian patients with 8q23–q24 deletions, two of whom do not involve *TRPS1*, focusing on clinical and genetic differences, and seeks to contribute to the current understanding of the molecular mechanisms underlying this syndrome.

## 2. Results

### 2.1. Patient 1

The first patient is a 16-year-old male who presented with a constellation of clinical features which include facial dysmorphism (hypertelorism, enophthalmos, low-set ears, and midface retrusion), multiple osteochondromas affecting the humeri, femora, and left tibia, along with skeletal anomalies such as brachydactyly, deformed fingers, shortened lower limbs and toes, syndactyly of toes II–III, and down-sloping shoulders. Additionally, gynecomastia was noted. Neurological assessment revealed a mild intellectual disability and motor tics involving the lips.

aCGH (array Comparative Genomic Hybridization) testing identified a 7.46 Mb deletion within the 8q24.11–q24.13 region. The detected copy number variation (CNV) encompasses 79 genes, notably including only one of the three primary TRPS II-associated genes, *EXT1*.

### 2.2. Patient 2

Our second patient is an 8-year-old girl who presented with multiple osteochondromas. She was born at 39 weeks of gestation to non-consanguineous parents following an uneventful pregnancy. Family history is largely unremarkable, with the exception of her mother, who also exhibits osteochondromas in both heels and the left foot. Her neurodevelopmental milestones were achieved normally. The initial osteochondroma was observed on the right scapula at one year of age. At the time of examination, she presented with osteochondromas located on the right humerus, lower right femur, and upper left tibia. Additional findings included mild facial dysmorphism characterized by a V-shaped frontal hairline, facial asymmetry, thick and broad eyebrows, partial bilateral upper eyelid coloboma, Brushfield spots, large prominent ears, a broad nasal ridge, dental anomalies, and a supernumerary tooth. Other features comprised dystrophic nails, upper limbs joint laxity, several psoriasiform lesions, and mild expressive language difficulties.

The phenotype presented by this patient initially prompted investigation for variants in genes associated with hereditary multiple osteochondromas. However, an initial TruSight One NGS (Next Generation Sequencing) panel yielded no pathogenic findings, as did a standard karyotype analysis (46,XX). The MLPA analysis returned a positive result, identifying a deletion in the 8q24.11 region that encompassed the *EIF3H* and *EXT1* genes. This result was confirmed through microarray, which detected a 3.32 Mb 8q23.3–8q24.12 deletion (23 genes, including *RAD21* and *EXT1*).

### 2.3. Patient 3

Patient 3 is an 18-year-old girl with no significant family or prenatal history; after birth, a vaginal atresia was observed, for which she underwent surgical correction at three days of age. She had an early neurodevelopmental delay (head control achieved at one year, ambulation at two years, and speech development at two and a half years), with normal results later in school. Physical examination revealed a mild dyslalia, myopia, decreased body weight, microcephaly, facial dysmorphism (sparse hair, macrotia, posteriorly rotated ears, midface retrusion, prominent nasal tip, long philtrum, thin upper lip, and thick lower lip), pectus excavatum, joint hypermobility, dolichostenomelia, equinovarus, arachnodactyly, and cone-shaped epiphyses of the hand phalanges.

Similarly with patient 2, MLPA identified a deletion in the 8q23.3–q24.11 region (*TRPS1* and *EIF3H* genes), with a microarray analysis establishing the genomic coordinates and size (2.09 Mb, 12 genes). This is the only patient from our group with a deletion encompassing *TRPS1*. *RAD21* is also part of the lost region.

**Figure 1:**
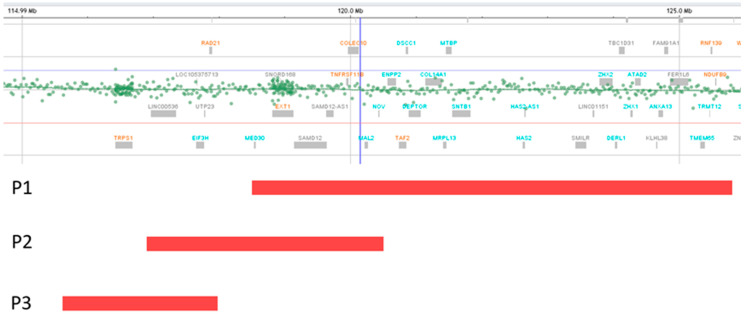
Deletion representations (red bands) for patients 1, 2, and 3. The deletion of P2 partially overlaps with P1 and P3 deletions. Deletions of P1 and P3 do not overlap.

The identified deletions are illustrated in Figure 1. Table 1 highlights the presence of TRPS II clinical aspects [1] in our patients. The identified CNVs are summarized in Table 2.

## 3. Discussion

The clinical variability previously described in patients with TRPS II is also observed in these cases [3,17]. All three patients presented facial dysmorphic features, but only patients 2 and 3 showed elements more frequently associated with TRPS I and II (details in Table 1) [1]. Particularly, the medial eyebrow of the second patient was denser and wider than the lateral eyebrow, an aspect which is considered common in TRPS [1,2,3,17].

Regarding the ectodermal anomalies, affected individuals usually have fine, sparse hair, notably in the frontotemporal region [18,19]. Sparse hair was only observed in patient 3, while patient 2 presented a V-shaped frontal hairline, along with dystrophic nails and a supernumerary tooth.

All three patients had skeletal abnormalities, with multiple exostoses (probably osteochondromas) seen only in patients 1 and 2. Since both of them have deletions encompassing the *EXT1* gene, the presence of these elements is expected (considering the association between *EXT1* and hereditary multiple osteochondromas), as is the absence of such features in patient 3, who carries a deletion sparing *EXT1* [20].

While the motor delays are considered to be secondary to the skeletal anomalies, more than half of individuals with TRPS II have mild-to-moderate intellectual disability [3,17]. In our group, only patient 1 presented mild intellectual disability, while patients 2 and 3 had a mild expressive language impairment, and a language delay with mild dyslalia, respectively.

Although vaginal atresia (noted in patient 3) is an uncommon manifestation, it has been previously observed in other TRPS II patients [21].

The milder phenotype observed in patient 2 may be partially attributed to the absence of *TRPS1* from the deleted region, as indicated by both the MLPA probe specific to this gene and the microarray result.

Similarly, *TRPS1* and *RAD21* are not encompassed within the deletion identified in patient 1, as the affected genomic segment commences more distally from the centromere compared to the commonly deleted region [1]. However, this deletion is significantly larger compared to the ones observed in patients 2 and 3, containing an important number of genes that could contribute to the phenotype (Table 2).

The minimal critical region responsible for TRPS II seems to be around 3.2 Mb, with no correlation with the severity of the intellectual disability [17,22]. *TRPS1* and *EXT1* are considered responsible for most of the clinical manifestations [22], with other features (intellectual disability, epilepsy, and growth hormone deficiency) more likely to be present as a result of a larger deletion encompassing more genes [3,23,24]. As for *RAD21*, a loss of this gene might be related to the more severe clinical aspects, particularly the cognitive impairment. Interestingly, features seen in patients with isolated *RAD21* haploinsufficiency have not been observed in TRPS II [1], suggesting that further modifiers and interactions contribute to these features.

*TRPS1*, *RAD21*, and *EXT1* individual gene–phenotype correlations are summarized in Table 3.

To date, to our knowledge, only seven patients harboring 8q23–q24 distal deletions not including *TRPS1* have been previously reported [5,12,13,14,15,16], with these cases usually referred to as Langer–Giedion-like, mixed LGS and Cornelia de Lange syndrome, overlapping or atypical phenotypes (Table 4). A high clinical variability is also observed in these patients (most likely related to the CNV sizes and gene contents), comprising dysmorphic features, skeletal anomalies, exostoses, neurodevelopmental delay, seizures, autism spectrum disorders, and premature puberty.

Patients 1 and 2 from this report, who also carry deletions distal to *TRPS1*, present manifestations in keeping with the literature (Table 4). Since only the deletion found in patient 3 involves *TRPS1*, and its size is reduced compared to the minimal critical region of TRPS II, this loss is expected to be more proximal. Accordingly, it overlaps only limited segments of previously reported deletions that spare *TRPS1* (Table 4), as well as a small fragment of the deletion identified in patient 2 (Figure 1). In contrast, although uncommon, the deletion of patient 3 does not overlap at all with that of patient 1 (Figure 1), as patient 1 carries the most distal 8q24.11–q24.13 deletion not encompassing *TRPS1* reported so far.

Interestingly, some authors mention that, although these deletions are distal to *TRPS1*, its expression could be affected if the breakpoint is located in its proximity, possibly affecting an unknown *TRPS1* gene regulator [13,14]. In such instances, the alteration of the respective gene expression would be less severe compared to the genes from the deleted region [25], further contributing to the clinical variability. More distal CNVs seem to lead to a reduced clinical overlap with LGS, supporting the hypothesis of a *TRPS1* expression regulatory sequence in the deleted region [14]. Furthermore, a report of a patient carrying an incompletely balanced translocation, t(2;8)(p16.1;q23.3), and presenting LGS clinical features, has suggested the presence of a possible regulatory element at 300 kb from *TRPS1* 5′ end [26]. Other possible mechanisms previously mentioned by other authors that could explain the overlapping TRPS II phenotype include the involvement of un undescribed gene [13], and a silencing effect on *TRPS1* determined by the chromosomal imbalance [14].

Apart from the genes classically associated with TRPS II, other genes considered of interest in CNVs distal to *TRPS1* include *ENPP2* and *NOV* (expressed in the adrenal gland) [14], and *KCNQ3*, associated with epilepsy [23]. However, many clinical features seen in patients with deletions distal to *TRPS1* are yet to be correlated to a specific gene, requiring further studies.

While these deletions can appear de novo, they can also be a result of balanced parental insertions involving the 8q23–q24 region [27]. Moreover, TRPS II can be caused by mosaic deletions, sometimes with a low degree in blood lymphocytes [28]. Therefore, the possibility of tissue–specific genetic testing should be taken into account if there is a negative blood test result, but the phenotype is highly suggestive.

Although both genetic testing methods, MLPA and microarray, are able to confirm the diagnosis, the lack of accuracy implied by the MLPA method in terms of establishing CNVs sizes, coordinates, and gene contents, represents an issue when comparing genotypes and trying to corelate with clinical aspects. The MLPA probemix used in our patients, P064 for Microdeletion Syndromes-1B, contains only three probes for the TRPS II region: one probe for *TRPS1* (8q23.3), one probe for *EIF3H* (8q24.11), and one probe for *EXT1* (8q24.11); there are no probes for *RAD21* (8q24.11), the other main gene associated with TRPS II [1].

Although techniques such as whole exome sequencing (WES) are becoming more prominent, offering the possibility to also detect CNVs [29], access in developing countries, such as Romania, is still hindered by several factors [30]. Therefore, the choice of methodology for detecting copy number variations was, in the presented cases, determined by the clinical and infrastructural contexts. For all three patients in this study, microarray analysis was the preferred diagnostic approach. However, due to the unavailability of microarray in our center at the time of patients 2 and 3 referral, MLPA (for microdeletions) was performed as an alternative, with microarrays also performed at a later time for confirmation and a better description of the deleted regions. Both techniques present distinct advantages and disadvantages. MLPA offers the benefits of fast turnaround time results and reduced costs, making it a highly suitable option when a specific MLPA kit is available for a suggestive phenotype. However, microarray provides genomic coordinates and comprehensive gene contents for identified CNVs, serving as a robust diagnostic tool, particularly in cases lacking clear clinical suspicion. Given these considerations, the utility of an aCGH analysis for patients 2 and 3 was clear, and the decision to offer this test was justified from both research and medical perspectives, as the gene contents of the deleted regions might be informative in terms of evolution and prognosis.

Only patient 2 presented a family history suggestive of TRPS II; specifically, her mother also had multiple osteochondromas. For the purpose of family members’ diagnosis, MLPA seems more suitable, as the main goal would be the confirmation of the CNV. However, since the deletion could be a result of a parental structural chromosomal anomaly, such as an insertion [27], further testing options (karyotype, and aCGH) could be considered in order to be able to provide more accurate genetic counselling if necessary.

The main limitations in our study are represented by the small number of patients that were evaluated, and the clinical and genetic heterogeneity of the results.

## 4. Materials and Methods

The three patients included in this study are unrelated and were genetically diagnosed in the Regional Centre for Medical Genetics Dolj, Craiova, Romania. The diagnostic process varied for each patient, with patients 1 and 3 also undergoing initial clinical evaluations in different centers in Bucharest.

Peripheral venous blood samples were available for all patients; DNA extraction was performed using the Wizard^®^ Genomic DNA Purification Kit from Promega (Madison, WI, USA). Subsequently, patient 1 underwent array comparative genomic hybridization testing on a SurePrint G3 CGH ISCA v2 4 × 180K slide from Agilent (Santa Clara, CA, USA). Patients 2 and 3 were initially tested through Multiplex Ligation-dependent Probe Amplification (probemix P064 for Microdeletion Syndromes-1B from MRC Holland, Amsterdam, The Netherlands), followed by a microarray evaluation on the Infinium CytoSNP-850K BeadChip v1.4 kit from Illumina (San Diego, CA, USA).

Particularly, patient 2 had previously undergone Next-Generation Sequencing TruSight One (TSO) panel evaluation and conventional karyotype testing.

## 5. Conclusions

Trichorhinophalangeal syndrome type II and 8q23–q24 deletion overlapping syndromes are rare disorders characterized by significant clinical variability, which is primarily influenced by the gene content of the underlying chromosomal deletion. Our data from three Romanian cases, two of whom presenting deletions sparing *TRPS1*, supports previously established correlations; however, further reports and studies are necessary in order to identify new mechanisms and associations, and to formulate a clearer nomenclature.

## Figures and Tables

**Table 1:** Clinical aspects present in our patients with overlapping TRPS II [1].

Sign/Symptom		Patient 1	Patient 2	Patient 3
Characteristic facial features	Large nose			
	Broad nasal ridge		+	
	Broad nasal tip			+
	Underdeveloped nasal alae			
	Broad nasal septum			
	Thick and broad eyebrows		+	
	Long philtrum			+
	Thin upper vermillion			+
	Large prominent ears		+	+
Ectodermal features	Hair anomalies		+	+
	Dystrophic nails		+	
	Hypoplastic breasts			
Skeletal features	Short stature			
	Short feet	+		
	Brachydactyly	+		
	Ulnar or radial deviation of the fingers			
	Hip dysplasia			
	Multiple osteochondromas	+	+	
Intellectual disability		+ (mild)		

**Table 2:** CNVs identified in the three patients.

	MLPA Result	Microarray Result			
		CNV Location	CNV Size	CNV Coordinates	Genes (OMIM Morbid)
Patient 1	not performed	del 8q24.11–q24.13	7.46 Mb	118,411,534–125,872,913 (GRCh37)	*EXT1*, *SAMD12*, *TNFRSF11B*, *COLEC10*, *TAF2*, *RNF139*, *NDUFB9*
Patient 2	del 8q24.11 (*EIF3H*, *EXT1*)	del 8q23.3–8q24.12	3.32 Mb	116,156,487–119,477,168 (GRCh38)	*RAD21*, *SLC30A8*, *EXT1*, *SAMD12*, *TNFRSF11B*, *COLEC10*
Patient 3	del 8q23.3–q24.11 (*TRPS1*, *EIF3H*)	del 8q23.3–8q24.11	2.09 Mb	115,042,661–117,138,754 (GRCh38)	*TRPS1*, *RAD21*, *SLC30A8*

**Table 3:** Main individual gene–phenotype correlations in the 8q23–q24 region relevant for the reported patients [1].

Gene Symbol	Locus	Individual Disease Association	Phenotypes
***TRPS1*** **(OMIM 604386)**	8q23.3	Trichorhinophalangeal syndrome, type I (OMIM 190350)	Characteristic facial features (large nose, broad nasal ridge and tip, underdeveloped alae, broad nasal septum, thick and broad medial eyebrows, long philtrum, thin upper lip, and large prominent ears). Ectodermal features (sparse hair, dystrophic nails, and hypoplastic breasts). Skeletal anomalies (short stature, brachydactyly, cone-shaped epiphyses, ulnar or radial deviation of the fingers, short feet, hip dysplasia, and early osteoarthritis).
***RAD21*** **(OMIM 606462)**	8q24.11	Cornelia de Lange syndrome 4 (OMIM 614701)	Intellectual disability; Elements specific to isolated *RAD21* deletions are yet to be reported in TRPS II patients.
***EXT1*** **(OMIM 608177)**	8q24.11	Exostoses, multiple, type 1 (OMIM 133700)	Multiple osteochondromas.

**Table 4:** Characteristics of previously reported patients with deletions not involving *TRPS1*.

No.	Reference (Year)	Reported Deleted Interval	Key Clinical Features Reported
**1**	Wuyts et al. (2002) [12]	del 8q24; interval likely involving *EXT1* and additional distal genes (genetic testing performed through FISH and PCR microsatellite analysis)	Multiple exostoses, hypertrichosis, intellectual disability, epilepsy.
**2**	McBrien et al. (2008) [13]	del 8q24.11; 117,725,989–119,191,934; 1.46 Mb	Exostosis, mild developmental delay, facial/skeletal features overlapping LGS; authors suggest a functional disturbance of *TRPS1* as an effect of the CNV.
**3**	Pereza et al. (2012) [14]	del 8q23.3-8q24.13; 116,921,245–124,442,990; 7.5 Mb	LGS-like phenotype, multiple exostoses, characteristic facial features, mild developmental delay, premature adrenarche.
**4**	Deardoff et al. (2012) [5], patient 1	del 8q24.11-8q24.12; 117,708,713–121,024,193; 3.3 Mb	Exostoses, dysmorphic features, skeletal anomalies.
**5**	Deardoff et al. (2012) [5], patient 4	del 8q23.3-8q24.11; 116,950,003–118,944,486; 2 Mb	Exostoses, dysmorphic features, neurodevelopmental delay.
**6**	Herrero-García et al. (2019) [15]	del 8q23.3-8q24.1; 116,915,114–119,171,074; 2.3 Mb	Mixed phenotype with features of LGS (exostoses, dysmorphic features) and Cornelia-de-Lange-like signs, neurodevelopmental delay, premature adrenarche; authors use the term mixed/overlapping.
**7**	Kim et al. (2021) [16]	del 8q24.11-8q24.13, 118,625,768–124,169,620; 5.5 Mb	Exostoses, dysmorphic features, intellectual disability, autism spectrum disorder.

## Data Availability

The original contributions presented in this study are included in the article. Further inquiries can be directed to the corresponding author(s).

## Abbreviations

The following abbreviations are used in this manuscript:

TRPS	Trichorhinophalangeal syndrome
LGS	Langer-Giedion syndrome
CIPO	chronic intestinal pseudo-obstruction
OMIM	online Mendelian Inheritance in Man
aCGH	array Comparative Genomic Hybridization
MLPA	Multiplex Ligation-Dependent Probe Amplification
CNV	Copy Number Variation
WES	Whole Exome Sequencing
